# Editorial Note: A Differential Effect of *E*. *coli* Toxin-Antitoxin Systems on Cell Death in Liquid Media and Biofilm Formation

**DOI:** 10.1371/journal.pone.0291587

**Published:** 2023-09-13

**Authors:** The PLOS ONE Editors

Readers have raised concerns about quantitative results published in bar graphs in Figs 2–3, S3-S7, and S9 of this article [1, 2]. Specifically, it was noted that the bars and axes demarcations are not consistently spaced within the bar graphs; there are some misalignments between error bars and data points, or between axes and their demarcations; some bars have lower edges that appear slightly above or below the X axis line; and some bars are similar to each other.

Also, in Figs 2A, 2B, 2C, S3A, S3B, S3C, S3D, S5B, S5C, and S6B, some data annotations (values) on the graphs do not agree with bars’ alignment with values on the Y axis. The authors stated that the data annotations within the graphs (i.e., above the bars) provide the correct results.

In addition, in Fig S5C, the data values on top of the bars are tenfold higher than the scale on the Y axis. The authors stated that the measured population size was 10^6^ for stained population size and the Y axis for Fig S5C should be labelled, ‘Absolute number of dead cells (x 10^6^)’.

The authors stand by the results and commented that these issues are due to the method(s) used to prepare the graphs. They stated that they used an early illustrator program into which they copied the values of calculated results (means and standard deviations) of three independent experiments for plotting on electronic millimeter grid paper [3].

The authors stated that the original raw data underlying the graphs of concern are not fully available at this time given the time elapsed. In light of this issue, PLOS has been unable to verify the results and the article does not currently comply in full with the applicable version of the PLOS Data Availability policy*. Nevertheless, the editors consider that the explanation provided by the authors sufficiently clarifies how the graphs were generated.

*The PLOS Data Availability policy was updated in 2014. The former policy (which was in place in 2008–09, [4]) still required authors to make data integral to an article’s findings freely available without restriction. The pre-2014 policy advised that data should be provided in Supporting Information files, a public repository, or upon request, but it did not strictly require publication or deposition of all quantitative data.
